# Association of Diabetes Mellitus and COVID-19-Related Pancreatic and Biliary Inflammatory Diseases

**DOI:** 10.3390/diagnostics16060903

**Published:** 2026-03-18

**Authors:** Chi-Yi Peng, Yu-Fong Lin, Wai-Keung Chow, Yen-Chun Peng, Cheng-Hung Lai

**Affiliations:** 1 Department of Veterinary Medicine, National Chung-Hsing University, Taichung 402202, Taiwan; pcynick@gmail.com; 2 Division of Gastroenterology, Department of Internal Medicine, Taichung Veterans General Hospital, Taichung 407219, Taiwan; pychunppp@gmail.com; 3 Division of Gastroenterology, Department of Internal Medicine, Taichung Veterans General Hospital Puli Branch, Nantou 545402, Taiwan; 4 School of Medicine, National Yang Ming Chiao Tung University, Taipei 112304, Taiwan; 5 Department of Post-Baccalaureate Medicine, National Chung-Hsing University, Taichung 402202, Taiwan

**Keywords:** COVID-19, type 2 diabetes mellitus (T2DM), pancreatitis, biliary tract ections, cholecystitis, mortality

## Abstract

**Background/Objectives:** The COVID-19 pandemic has brought about significant clinical challenges in regard to digestive systems, as well as causing complications such as pancreatitis and biliary infections. Whether diabetes mellitus (DM) contributes to both an increased risk for these complications and mortality amongst COVID-19 patients remains to be investigated. This study aimed to illuminate any possible outcomes, including pancreatitis, cholangitis, cholecystitis and all-cause mortality, among COVID-19 patients with and without pre-existing type 2 diabetes mellitus (T2DM), using real-world data taken from a multinational electronic health record database. **Methods:** A retrospective cohort study based upon data taken from the database of the TriNetX Global Collaborative Network was conducted. We included patients from the database who had been diagnosed with COVID-19 from January 2020 to December 2023. Enrolled subjects were divided into two cohorts: COVID-19 patients with pre-existing T2DM who had had at least two medical visits, and those without T2DM. Propensity score matching was performed using 68 baseline variables. Outcomes were evaluated within 90 days following COVID-19 diagnosis, with patients with prior relevant diagnoses being excluded. Risk analyses, Kaplan–Meier survival estimates, and hazard ratios were calculated as the outcomes. **Results:** The incidence of acute pancreatitis was significantly higher in the DM+ group when compared to the DM– group (Hazard ratio (HR) = 1.307; 95% confidence interval (CI) 1.048–1.630, *p* = 0.017) and mortality (HR = 1.141; 95% CI 1.102–1.181, *p* < 0.05) by Kaplan–Meier analysis. Risk of cholecystitis (HR = 1.264; 95% CI 1.042–1.533, *p* = 0.017) was borderline increased, and cholangitis was not significant (HR 0.847, 95% CI 0.583–1.230) **Conclusions:** In COVID-19 patients, pre-existing T2DM is independently associated with increased risks of acute pancreatitis and mortality.

## 1. Introduction

Severe acute respiratory syndrome Coronavirus 2 (SARS-CoV-2) is a positive-sense single-stranded RNA virus that caused Coronavirus disease 2019 (COVID-19) beginning in late 2019 [1]. Since its outbreak, the COVID-19 pandemic has evolved into a sustained global health concern worldwide, exerting a profound impact on healthcare systems while also causing extensive morbidity and mortality across all populations. Although COVID-19 is primarily recognized for its pulmonary manifestations, the systemic nature of the disease has become increasingly evident, with extrapulmonary involvement being seen [2,3,4].

Meta-analysis of international data has disclosed that the gastrointestinal manifestation of COVID-19 include nausea, vomiting, diarrhea and abdominal pain, although all were found to be less common (<10%), and not specific [5,6]. There is proposed evidence that certain digestive symptoms may be related to disease severity [7]. Among these, gastrointestinal system and liver function impairment were both mentioned. Additionally, and more specifically, the pancreas and biliary tract have each emerged as areas of interest due to reports of acute pancreatitis and cholangiopathy in infected individuals, with some data suggesting that pancreatobiliary complications are associated with more severe disease status [8,9,10,11,12].

Diabetes mellitus (DM) has been consistently implicated as a risk factor for adverse outcomes in patients diagnosed with COVID-19 [13]. The underlying mechanisms are thought to involve immune dysregulation, chronic inflammation, and vascular dysfunction, all of which may predispose individuals with diabetes to more severe disease [14]. The role of diabetes in modulating the risk or severity of pancreatic and biliary events during or after COVID-19 infection remains poorly defined [15]. Understanding whether diabetes confers susceptibility to COVID-19-related pancreatobiliary complications could yield important clinical implications [16].

Diabetes is also considered to carry the risk of acute pancreatitis, biliary tract inflammation, or infection [17,18]. Thus, it could be considered a cholangiopathy rather than traditional truly acute cholangitis [11]. The same situation also occurs in gallbladder inflammation, where COVID-19 causes acalculous cholecystitis rather than infectious calculous cholecystitis.

Diabetes mellitus (DM) may be implicated in the development or exacerbation of acute pancreatitis, cholangiopathy or cholangitis, and cholecystitis. However, the complex interplay that exists between COVID-19, diabetes, and pancreatic or biliary tract inflammation remains insufficiently explored. It is plausible that DM contributes to the pathogenesis or worsening of the clinical course of these conditions through either direct metabolic effects or indirect immune-mediated mechanisms. Elucidating the role of diabetes in COVID-19-associated pancreatic and biliary complications is therefore of clinical relevance and certainly warrants further investigation.

In the present study, we aimed to investigate the association between diabetes mellitus and the occurrence of acute pancreatitis, cholangitis or cholangiopathy, and cholecystitis, as well as the related mortality, in patients following COVID-19 infection by utilizing data taken from the TriNetX global health research network.

## 2. Materials and Methods

### 2.1. Data Source

We conducted a retrospective, comparative cohort study using data taken from the TriNetX Global Collaborative Network (Cambridge, Massachusetts, USA), a federated platform comprising 147 healthcare organizations worldwide. The network provides a platform for accessing de-identified electronic medical records, including structured clinical data such as diagnoses, procedures, medications, laboratory results, demographics and mortality outcomes. An internal analysis using the TriNetX system (version 4.x) can be employed to both collect clinical data and perform statistical analyses of clinical outcomes. As the role of real-world data in healthcare continues to expand, TriNetX stands out as a critical tool that complements traditional clinical trials, bridging the gap between controlled research environments and real-world practice. This platform provides a comprehensive analysis of the methodologies and applications highlighting its potential contribution to the advancement of patient care and medical outcomes [19]. Analyses investigating the relationship between COVID-19, diabetes and psychological diseases have also been conducted using the TriNetX database [20].

### 2.2. Study Design

Two cohorts were defined for analysis in this study: Cohort 1 (*n* = 116,192) consisted of adult patients (≥20 years) diagnosed with COVID-19 who had a prior history of type 2 diabetes mellitus (T2DM) (DM+), while Cohort 2 (*n* = 116,192) included matched patients with COVID-19 but without T2DM (DM−), identified via propensity score matching. The inclusion criteria for both cohorts required laboratory-confirmed SARS-CoV-2 RNA positivity and a minimum of two clinical visits. The index date was defined as the first COVID-19 diagnosis meeting all criteria during the period of 1 January 2020 to 31 December 2023, with outcomes being assessed during a fixed 90-day window beginning one day after the index event.

The study employed the TriNetX built-in analytical functions to perform risk analysis, Kaplan–Meier survival analysis, and number-of-instances analysis for four clinical outcomes: acute pancreatitis, cholangitis, cholecystitis and all-cause mortality. Patients with an outcome prior to the time window were excluded in order to reduce bias. Matching between cohorts was based on age, gender, BMI, race, and extensive baseline characteristics, including comorbidities of overweight, obesity and other hyperalimentation; hypertensive diseases; disorders of the thyroid gland; diseases of the esophagus, stomach and duodenum; diseases of the liver; chronic lower respiratory diseases; diseases of the arteries, arterioles and capillaries; ischemic heart disease; osteoarthritis; spondylopathies; other soft tissue disorders; other joint disorders; other diseases of the respiratory system; influenza and pneumonia; other diseases of the intestine; disorders of the gallbladder, biliary tract, and pancreas; diseases of the nervous system; neoplasms; external causes of morbidity; laboratory values of sodium, potassium, creatinine, calcium, phosphate, leukocytes, erythrocytes, hemoglobin, platelets, neutrophils, alanine aminotransferase, aspartate aminotransferase, alkaline phosphatase, gamma-glutamyltransferase, lactate dehydrogenase, bilirubin, albumin, prothrombintime, triglycerides, cholesterol, Hba1c, thyroglobulin, serum/plasma/blood chloride, serum/plasma/blood bicarbonate, serum/plasma/blood urea nitrogen, serum/plasma/blood glucose, and serum/plasma/blood magnesium; and medication exposures including hormones/synthetics/modifiers, cardiovascular medications, antiarrhythmics, anti-lipemic agents, betablockers and related agents, diuretics, calcium channel blockers, ACE inhibitors, other antihypertensives, antianginals, angiotensin II inhibitors, respiratory agents (other), antiasthma/bronchodilators, antitussives/expectorants, systemic decongestants, mucolytics, and antacids. All were compared both pre- and post-matching to ensure balance. This analytical setup enabled the rigorous assessment of whether diabetes modifies the risk of pancreaticobiliary complications and mortality in COVID-19 patients during the acute phase of infection.

### 2.3. Cohort Definitions

The two patient cohorts were defined based on their COVID-19 status and history of T2DM, using data from 1 January 2020 to 31 December 2023. All patients were aged ≥20 years at the time of COVID-19 diagnosis, confirmed by both ICD-10 code (U07.1) and positive SARS-CoV-2 RNA laboratory testing. Cohort 1 (DM+) included patients with a documented diagnosis of T2DM (ICD-10: E11) prior to or on the same day as the COVID-19 diagnosis, with ≥2 clinical encounters in the database. Cohort 2 (DM–) included patients with confirmed COVID-19 but without any documented diagnosis of T2DM, also with ≥2 clinical encounters. Patients with prior diagnoses of study outcomes (acute pancreatitis, cholangitis, cholecystitis or death) before the index date were excluded to reduce confounding from any pre-existing conditions.

The analysis process included two main steps: defining the cohorts through query criteria, and then setting up and running the analysis. Setting up the analysis required defining the index event, outcomes criteria, and the time frame. In the present study, we defined the basic data time frame as COVID-19 diagnosis being from 5 years ago to 1 day ago. The outcomes were collected within 90 days after the index date of COVID-19 being diagnosed. Comparing the outcomes involved the use of four analyses: Measures of Association, Survival, Number of Instances and Laboratory Result Distribution. 

### 2.4. Index Event and Time Window

The index event was defined as the earliest date on which a patient met the inclusion criteria for COVID-19 infection, confirmed through both diagnostic code (e.g., ICD-10 code U07.1) and a laboratory-verified positive test for SARS-CoV-2. This definition ensured there was a consistent identification of cases across institutions within the dataset.

The outcome assessment window began on Day 1 following the index date to capture only incident events occurring after the confirmed infection as well as to avoid potential immortal time bias. Patients were followed up for a period of 90 days, which was selected as a clinically meaningful interval for monitoring early post-infection complications. The outcomes of interest included acute pancreatitis, cholangitis, cholecystitis and all-cause mortality, reflecting potential hepatobiliary and pancreatic involvement following COVID-19. These outcomes were chosen based on emerging clinical evidence suggesting that SARS-CoV-2 infection may contribute to inflammatory processes in the gastrointestinal and hepatopancreatobiliary systems, particularly in patients with underlying metabolic or immune-related vulnerabilities.

### 2.5. Propensity Score Matching

To minimize baseline confounding and enhance comparability between groups, 1:1 ratio propensity score matching was conducted using a nearest-neighbor algorithm without replacement and a caliper width of 0.01 of the standard deviation of the logit of the propensity score. Propensity score matching was performed using 68 baseline variables, including demographics (age, gender, race, body mass index), comorbidities, laboratory parameters, and medication exposures, to ensure adequate control for any potential confounders. Following this matching, the baseline characteristics of the cohorts were re-evaluated to assess covariate balance. Standardized mean differences were calculated for each variable, with values less than 0.1 indicating an acceptable level of balance between the two groups. Furthermore, characteristics of the cohorts that are balanced using propensity score matching are also included in the Propensity Score Matching section. We display the data both before and after matching in our study groups (Table 1).

### 2.6. Outcome Measures

We used ICD-10 diagnosis to define the outcome measure. The primary outcomes of pancreas and biliary events in COVID-19 infected subjects with or without T2DM were acute pancreatitis (ICD-10: K85), cholangitis (ICD-10: K83.0), cholecystitis (ICD-10: K81) and all-cause mortality (based on death status in the demographic data). In this study, the term ‘cholangitis’ refers to cases identified by ICD-10 codes. The database does not allow for differentiation of specific clinical subtypes, such as cholangiopathy. The events were collected within 90 days after COVID-19 had been diagnosed.

Each clinical outcome was evaluated by time-to-event (survival) analysis, which was conducted using Kaplan–Meier survival curves to visualize outcome incidence over time. Log-rank tests were applied to assess statistical differences in survival distributions between cohorts. Hazard ratios (HRs) with 95% confidence intervals (CIs) were estimated using Cox proportional hazard models, with the proportional hazards assumption tested to validate model appropriateness. Event frequency analysis focused on the cumulative number of incident cases occurring within a defined 90-day follow-up period. To ensure an accurate estimation of incident (new-onset) events, patients with a documented prior diagnosis of the respective outcome prior to the index date were excluded. This multi-method framework allowed for robust comparisons between cohorts, accounting for both incidence proportions and timing of outcome development.

### 2.7. Statistical Analysis

All statistical analyses were performed using the built-in analytics suite of the TriNetX platform, which provides integrated tools for cohort selection, matching, and comparative outcome analysis. Between-group comparisons of outcome proportions were evaluated using two-sided z-tests to determine risk differences, while independent samples *t*-tests were employed for comparing continuous or frequency-based data, where appropriate. A two-tailed *p*-value of <0.05 was considered indicative of statistical significance for all hypothesis testing.

For time-to-event outcomes, Cox proportional hazard regression models were applied to estimate HRs and their corresponding 95% CIs. To verify the assumption of proportional hazards, Schoenfeld residuals were examined when available through the analytic interface. The robustness of the proportionality assumption was further supported by inspection of log-minus-log survival plots when residual-based diagnostics were not accessible. All analyses adhered to the standard methodological practices used in comparative effectiveness research. The proportional hazards assumption was met for acute pancreatitis (χ^2^ = 0.803, *p* = 0.370) and cholecystitis (χ^2^ = 0.601, *p* = 0.438). However, it was not satisfied for cholangitis (χ^2^ = 4.469, *p* = 0.035) and mortality (χ^2^ = 20.163, *p* < 0.001). We acknowledge this limitation in the revised manuscript and emphasize that the interpretation of hazard ratios for cholangitis and mortality should be performed with caution, as time-varying effects could not be modeled within the constraints of the TriNetX platform.

### 2.8. Ethics and Data Privacy

As this study used only de-identified, aggregated data obtained from the TriNetX network platform, it was exempt from any Institutional review board (IRB) approval under U.S. Department of Health and Human Services regulation 45 CFR 46.104(d). All data handling complied with HIPAA and GDPR standards for both patient privacy and data security. The present study was also approved by our institutional review board (No. CE24589B).

## 3. Results

A total of 837,683 patients were initially identified within the TriNetX database. Inclusion criteria required a confirmed diagnosis of COVID-19, defined by both a positive SARS-CoV-2 test and an ICD-10 diagnostic code for COVID-19. To ensure data quality and relevance for follow-up analyses, patients were further restricted to those aged 20 years or older who had had at least two documented healthcare visits during the study period. These criteria ensured reliable capture of clinical outcomes and adequate longitudinal follow-up.

**Figure 1:**
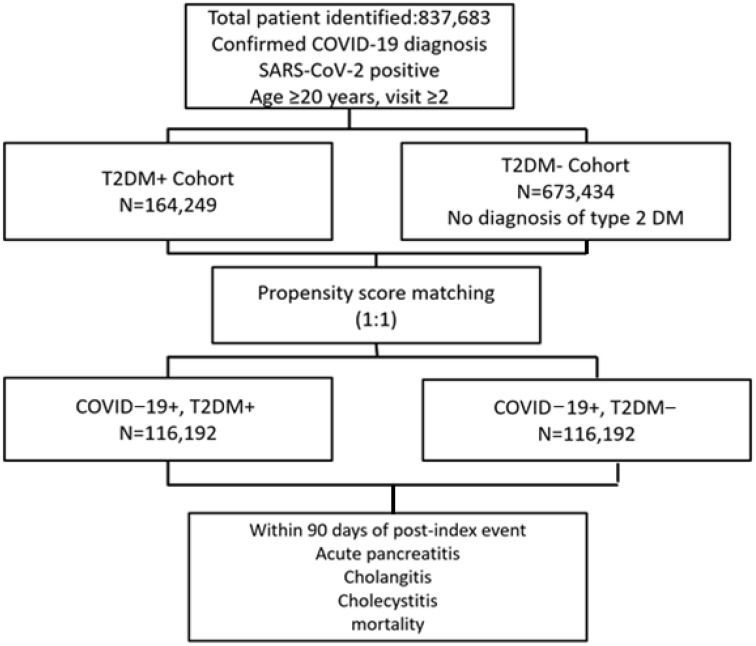
Diagram of patients identified and analyzed.

After propensity score matching, a total of 116,192 patients were included in each cohort: those with T2DM and COVID-19 infection (DM+), and those without diabetes (DM−). The mean follow-up period was approximately 82.974 days for the DM+ group and 82.595 days for the DM− group (Figure 1). Propensity score matching was performed using 68 baseline variables, including demographics (age, gender, race, body mass index), comorbidities, laboratory parameters, and medication exposures, to ensure adequate control for any potential confounders.

The clinical variables, including basic data, comorbidities and laboratory data, both before and after matching, are disclosed in Table 1. The demographic distribution and baseline clinical characteristics of the study cohorts were determined both before and after propensity score matching. Prior to matching, patients with T2DM (DM+) were found to be significantly older (mean age: 62.4 vs. 48.6 years) and more likely to be male (47.4% vs. 41.5%) when compared to those without diabetes (DM−), with standardized differences exceeding accepted thresholds. Comorbid conditions were also more prevalent in the DM+ group, including hypertension (85.9% vs. 31.5%), overweight status (51.6% vs. 20.1%), thyroid disorders (27.0% vs. 12.6%), and liver diseases (20.2% vs. 7.0%), all of which showed substantial imbalances (SD > 0.3). Laboratory differences reflected the metabolic burden in the DM+ group and included elevated creatinine, triglycerides and hemoglobin A1c, along with lower albumin levels. Following propensity score matching on the variables mentioned in the study design, balance was substantially improved across most variables, although residual differences persisted in key metabolic and inflammatory parameters, including triglycerides (SD = 0.321), hemoglobin A1c (SD = 0.957), and alkaline phosphatase (SD = 0.166). These residual imbalances underscore the systemic impact of diabetes on organ function and inflammation, even after demographic adjustment.

**Figure 2:**
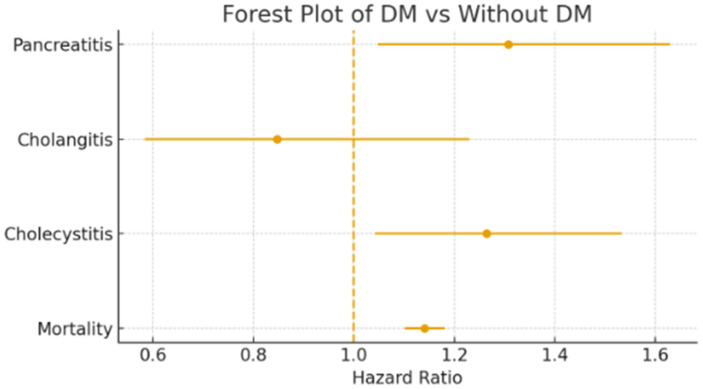
Forest plots of type 2 diabetes and COVD-19 for pancreas and biliary complications.

In Table 2 and Figure 2, we demonstrate the patients with diabetes and without diabetes in COVID-19-related pancreato-biliary complication and mortality.

A comparative analysis was performed between patients with and without DM to assess the incidence of acute pancreatitis, cholangitis, cholecystitis, and all-cause mortality. A total of 112,288 patients with DM and 113,169 patients without DM were included in analysis of pancreatitis. The incidence of pancreatitis was significantly higher in the DM group (181/112,288, 0.161%) compared to the non-DM group (139/113,169, 0.123%). The hazard ratio (HR) for pancreatitis among COVID-19 patients with DM was 1.307 (95% CI: 1.048–1.630; *p* = 0.017). This suggests that patients with pre-existing DM are substantially more susceptible to developing acute pancreatitis in the setting of SARS-CoV-2 infection. Regarding cholangitis, 51 cases were identified in the DM group (*n* = 115,543), while 60 cases occurred in the non-DM group (*n* = 115,610). The incidence rates were comparable between the groups (0.044% in with DM vs. 0.052% in without DM). The corresponding hazard ratio was 0.847 (95% CI: 0.583–1.230; *p* = 0.382), indicating no statistically significant association between DM status and risk of cholangitis in COVID-19 patients. In the case of cholecystitis, there were 234 events among 113,029 diabetic patients (0.207%) and 184 events among 112,837 non-diabetic patients (0.163%). Patients with DM had a moderately elevated risk of cholecystitis, with an HR of 1.264 (95% CI: 1.042–1.533; *p* = 0.017), suggesting a statistically significant risk. Mortality was also higher among COVID-19 patients with diabetes. Of 115,341 diabetic patients, 6845 deaths were recorded (5.9%), compared to 5982 deaths among 115,321 non-diabetic patients (5.2%). The mortality HR for COVID-19 with DM patients was 1.141 (95% CI: 1.102–1.181; *p* < 0.05), indicating a significantly elevated risk of death among COVID-19 patients with diabetes.

**Figure 3:**
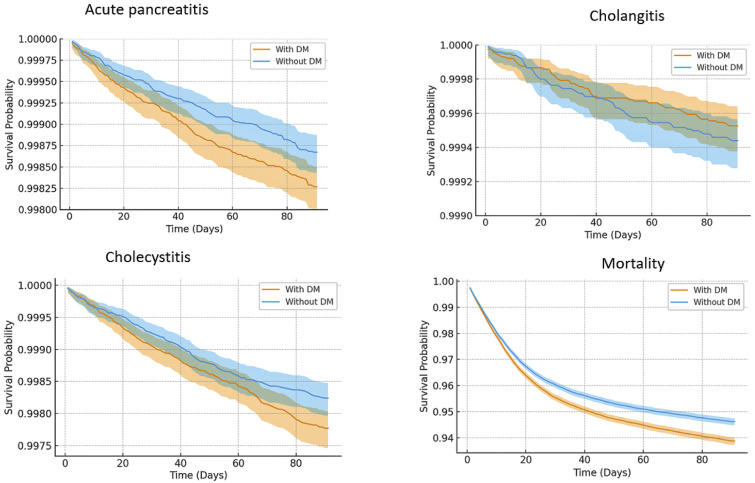
Kaplan–Meier survival curves comparing COVID-19 patients with and without T2DM for the outcomes of acute pancreatitis, cholangitis, cholecystitis, and all-cause mortality.

Figure 3 shows survival analyses conducted to compare pancreatobiliary outcomes in COVID-19 patients with and without DM. The Kaplan–Meier survival curve for acute pancreatitis, cholecystitis, and mortality revealed significantly lower survival probabilities in the DM cohort compared to the non-DM group, indicating a higher short-term risk of adverse events in diabetic patients.

The *p*-values in acute pancreatitis, cholangitis, cholecystitis, and mortality were 0.017, 0.382, 0.017, and <0.05 in patients with COVID-19 infection with or without type 2 diabetes mellitus, according to log-rank test.

In Figure 3, the *x*-axis represents time in days following COVID-19 diagnosis, and the *y*-axis indicates survival probability ranging from 0.93 to 1.00. Separate Kaplan–Meier curves are shown for patients with T2DM (DM, blue) and without DM (red), with shaded areas representing 95% confidence intervals. Hazard ratios (HR) with 95% confidence intervals (CI) were estimated using Cox proportional hazard models. Diagnoses were identified based on ICD-10 codes, and outcomes were assessed within the 90 days following COVID-19 diagnosis.

## 4. Discussion

This study evaluated the effect of T2DM on the incidence of pancreato-biliary complications and mortality in patients with COVID-19 during the COVID-19 pandemic between 2020 and 2023. The incidence of acute pancreatitis was significantly higher in the DM group, with a hazard ratio (HR) = 1.307 (95% confidence interval (CI) 1.048–1.630, *p* = 0.017), indicating increased susceptibility to pancreatitis in diabetic individuals. There was no significant difference observed in the incidence of cholangitis between DM vs. non-DM groups (HR 0.847, 95% CI 0.583–1.230; *p* = 0.382). For cholecystitis, COVID-19 patients with T2DM showed a modestly elevated risk (HR = 1.264; 95% CI 1.042–1.533, *p* = 0.017). All-cause mortality was significantly higher in the DM group (5.93% vs. 5.19%), with a hazard ratio of 1.141 (995% CI 1.102–1.181, *p* < 0.05). These findings suggest that diabetes is associated with an increased risk of pancreatitis, cholecystitis, and mortality among COVID-19 patients, but no significant risk of cholangitis.

DM is a worldwide metabolic disorder with increasing significance placed on the global healthcare burden [21]. Another concern regarding COVID-19 infection is the systemic consequences both during and after COVID-19 infection of what is called long COVID-19, which is still under investigation [3,22]. The systemic and long-term effects of DM have been under investigation for decades [23,24]. It is reasonable to hypothesize that the co-existence of COVID-19 and DM or COVID-19 infection in DM individuals would carry worse events. Therefore, it remains worthwhile that further research be performed with regard to the interplay of DM and COVID-19 infection, not only for the purpose of clinical care, but also for determining both systemic and long-term results in patients after COVID-19 infection. In the present study, we determined that DM is associated with pancreatic and biliary complications after COVID-19 infection.

Immune-related manifestations are being increasingly recognized in patients with COVID-19, with approximately 3000 cases reported worldwide, comprising more than 70 different systemic and organ-specific disorders [2]. Digestive systemic complications related to COVID-19 include gastrointestinal manifestations, which are seen more commonly [25], while less common are pancreatitis and biliary inflammation (cholangitis or cholangiopathy and acalculous cholecystitis) [10,11,12,26].

Pancreatobiliary complications may be divided into two categories: pancreatitis and biliary tract inflammations. COVID-19-related pancreatitis is generally considered a low-incidence occurrence, and is estimated at around 0.1–1.5%, a common health status that is more severe in certain subgroups [27,28,29]. Acute pancreatitis after COVID-19 infection may be related to direct or indirect injury by the SARS-CoV virus to its host [9]. Our results are the first to demonstrate that DM is a risk factor for acute pancreatitis, as compared to previously published data.

Biliary complication is a rare condition related to COVID-19 infection [10,11,12,26]. However, there is no data available with regard to any biliary complications due to COVID-19 infection, while liver dysfunction is estimated to occur in approximately 20% of COVID-19-infected individuals [30]. COVID-19 related cholangiopathy is more likely than infection of the biliary tract due to cholangitis. In the present study, we were unable to properly define cholangitis or cholangipathy, and therefore used cholangitis instead of cholangipathy. Thus, our results may be due to limited evidence. However, our results showed no significance in COVID-19 patients with or without DM regarding cholangitis. Although COVID-related cholangiopathy has been described as a distinct clinical syndrome characterized by persistent cholestasis and bile duct injury after severe COVID-19, our study could not specifically identify such cases. Therefore, we use the term ‘cholangitis’ to denote ICD-10-based diagnoses, while acknowledging that these are not clinically synonymous.

The same situation occurred with gallbladder inflammation, where cholecystitis is involved, and which could include acalculous cholecystitis and calculous cholecystitis. A previous report suggested that COVID-19 infection may be related to acute acalculous cholecystitis [26]. Our results demonstrated that COVID-19 patients with DM carry a risk of cholecystitis, but whether calculous or not could not be well defined.

The mechanism is complex and not well defined. Involvement of the pancreatic and biliary systems may be due to direct viral injury and/or an inflammatory immune response. Thus, pancreatic or biliary inflammation may be due to direct viral injury, systemic inflammation response, immune insults, or exacerbation of a pre-existing disease status [7,31]. Pancreato-biliary complications are rare but could often be seen in critically ill patients [6].

Diabetes exerts multifaceted effects on the host beyond just glycemic dysregulation. Chronic hyperglycemia compromises immune responses, rendering individuals more susceptible to infections while also impairing the healing process. In addition to metabolic disturbances, diabetes is increasingly recognized as a condition associated with immunological dysfunction. These immunologic alterations may be further modulated by underlying genetic risk factors that influence susceptibility to both autoimmunity and infection. Furthermore, individuals with diabetes often exhibit heightened vulnerability to physiological stressors, such as acute illness or inflammation, which can exacerbate disease severity and complicate clinical outcomes. Understanding these interrelated mechanisms remains crucial for the development of targeted interventions aimed at reducing infection-related morbidity and improving overall disease management in diabetic populations [32,33]. However, a retrospective cohort study performed at a tertiary center in Romania found that patients diagnosed with T2DM had a significantly higher risk of severe acute pancreatitis and were more likely to require intensive care unit admission. Nevertheless, the study underscores the still unclear and complex relationship between T2DM and pancreatitis after adjusting for cofounding factors. Thus, there remains a need for further prospective research in order to clarify their interplay and causal links [34].

There are several limitations in our study. First, the diagnosis of pancreatitis, cholangitis, and cholecystitis was based solely on ICD-10 codes, which could lead to misclassification. Second, the TriNetX database does not provide detailed clinical data such as COVID-19 disease severity, hospitalization or ICU course, BMI, antidiabetic therapy, steroid use, or immunosuppressive treatment in every enrolled subject. The absence of these variables limits adjustment for important confounders and may affect the robustness of our conclusions. Third, an important limitation of this study is the lack of a DM+/COVID− control group. However, the TriNetX database is limited in that it allows only two-group comparisons within a single analysis, which restricts this approach. Fourth, due to the limitations of ICD-10 coding for cholangiopathy, we used the cholangitis code as a proxy. This approach could lead to some degree of diagnostic ambiguity. Fifth, the TriNetX database is primarily designed for administrative use and allows only univariate comparisons, which limits the ability to adjust for potential confounders. In addition, as a multisite database, it may present heterogeneity in data collection and potentially have missing data. Therefore, future studies using more detailed and controlled datasets are warranted to help better validate these findings. Finally, Cox proportional hazard models were generated within the TriNetX platform after age- and gender-based propensity score matching, without adjustment for additional covariates. The proportional hazards assumption was fulfilled for acute pancreatitis and cholecystitis, but not for cholangitis and mortality, indicating potential time-varying effects that could not be modeled in this dataset. Therefore, the hazard ratio estimates for cholangitis and mortality should be interpreted with caution.

## 5. Conclusions

Our study defined the association between diabetes and COVID-19-related complications of acute pancreatitis and mortality. T2DM is definitely associated with risk of acute pancreatitis and mortality. COVID-19 infection has not yet been eradicated throughout the world, and therefore, for patients diagnosed with DM, it would be worthwhile to show more consideration for the conditions of pancreatitis and biliary inflammation. Finally, with regard to long COVID-19 care, diabetes as a direct or indirect mechanism would also be worthy of investigation.

## Figures and Tables

**Table 1:** Demographic data of study cohorts before and after matching.

	Before Matching				After Matching			
Variable	With T2DM (*N* = 164,249) *n* (%) or Mean ± SD	Without T2DM (*N* = 673,434) *n* (%) or Mean ± SD	*p*-Value	Std Diff	With T2DM (*N* = 116,192) *n* (%) or Mean ± SD	Without T2DM (*N* = 116,192) *n* (%) or Mean ± SD	*p*-Value	Std Diff
Demographics								
Age (mean ± SD)	62.4 ± 14.3	48.6 ± 18.2	<0.001	0.845	60.8 ± 14.8	61.0 ± 16.0	<0.001	0.016
Female (%)	52.5%	58.5%	<0.001	0.119	54.0%	54.1%	0.405	0.003
Male (%)	47.4%	41.5%	<0.001	0.120	46.0%	45.7%	0.223	0.005
White (%)	64.0%	68.3%	<0.001	0.090	66.7%	66.5%	0.291	0.004
Black (%)	20.9%	15.5%	<0.001	0.140	18.9%	19.1%	0.149	0.006
Asian (%)	4.3%	3.8%	<0.001	0.029	4.1%	4.1%	0.753	0.001
Hispanic/Latino (%)	11.9%	12.1%	0.023	0.006	11.5%	11.4%	0.725	0.001
Not Hispanic/Latino (%)	70.9%	68.8%	<0.001	0.046	71.1%	71.3%	0.300	0.004
Diagnosis								
Hypertensive diseases	85.9	31.5	<0.001	1.326	90	90.4	0.001	0.013
Overweight, obesity	51.6	20.1	<0.001	0.696	56.7	55.9	<0.001	0.017
Disorders of thyroid gland	27	12.6	<0.001	0.367	24.6	24.6	0.828	0.001
Ischemic heart disease	40.5	10.3	<0.001	0.74	46	45.4	0.009	0.011
Liver disease	20.2	7	<0.001	0.392	50.4	50	0.041	0.008
Gallbladder/biliary/pancreas disorders	15.4	6	<0.001	0.308	38	37.5	0.013	0.01
Intestinal disease	46	23.5	<0.001	0.486	31	30.3	<0.001	0.015
Influenza and pneumonia	35.7	17.1	<0.001	0.432	66.7	66.7	0.993	0
Nervous system diseases	72.4	41	<0.001	0.668	79.7	80.5	<0.001	0.018
Neoplasms	36.9	21.1	<0.001	0.355	58.9	59	0.695	0.002
Medication								
Cardiovascular drugs	92.9	56.6	<0.001	0.919	90	90.4	0.001	0.013
Antilipemic agents	67.7	18.3	<0.001	1.152	56.7	55.9	<0.001	0.017
Diuretics	53.2	17.3	<0.001	0.813	46	45.4	0.009	0.011
Beta-blockers	57.5	20.9	<0.001	0.808	50.4	50	0.041	0.008
Calcium channel blockers	43.8	13.8	<0.001	0.703	38	37.5	0.013	0.01
ACE inhibitors	38.3	9.9	<0.001	0.702	31	30.3	<0.001	0.015
Antiasthma/Bronchodilators	69.9	44.5	<0.001	0.531	66.7	66.7	0.993	0
Respiratory agents	83.3	52.5	<0.001	0.7	79.7	80.5	<0.001	0.018
Antacids	65.4	30	<0.001	0.759	58.9	59	0.695	0.002
Laboratory data								
Sodium	137.8 ± 3.8	138.6 ± 3.3	<0.001	0.215	137.8 ± 3.7	138.5 ± 3.6	<0.001	0.173
Potassium	4.2 ± 0.5	4.1 ± 0.5	<0.001	0.215	4.2 ± 0.5	4.1 ± 0.5	<0.001	0.13
Creatinine	1.5 ± 3.8	1.1 ± 3.8	<0.001	0.122	1.4 ± 3.8	1.2 ± 4.1	<0.001	0.044
Calcium	9.1 ± 0.7	9.2 ± 0.6	<0.001	0.124	9.1 ± 0.7	9.1 ± 0.7	<0.001	0.014
ALT (GPT)	30.2 ± 67.3	30.8 ± 68.4	<0.001	0.009	30.4 ± 63.3	31.5 ± 74.0	<0.001	0.016
AST (GOT)	33.4 ± 146.6	32.1 ± 109.5	<0.001	0.01	32.7 ± 143.4	35.0 ± 153.8	<0.001	0.016
ALP	94.6 ± 68.9	82.8 ± 52.5	<0.001	0.192	93.0 ± 66.8	87.7 ± 61.9	<0.001	0.081
GGT	131.3 ± 260.6	94.3 ± 213.8	<0.001	0.155	127.1 ± 264.2	112.1 ± 238.6	<0.001	0.06
Bilirubin, total	0.6 ± 0.8	0.6 ± 0.8	<0.001	0.014	0.6 ± 0.8	0.6 ± 0.9	<0.001	0.048
Albumin	3.7 ± 0.7	4.0 ± 0.6	<0.001	0.39	3.7 ± 0.6	3.8 ± 0.6	<0.001	0.097
HbA1c	7.3 ± 1.9	5.6 ± 0.9	<0.001	1.137	7.3 ± 2.0	5.7 ± 1.0	<0.001	1.02
Glucose	152.5 ± 74.7	105.0 ± 31.7	<0.001	0.827	152.5 ± 75.2	109.5 ± 35.1	<0.001	0.733
Cholesterol	160.6 ± 48.0	180.3 ± 42.3	<0.001	0.434	164.0 ± 47.8	172.6 ± 45.2	<0.001	0.185
Triglyceride	163.4 ± 146.0	126.6 ± 88.6	<0.001	0.305	164.2 ± 146.4	133.3 ± 92.1	<0.001	0.253
Urea nitrogen	22.4 ± 16.4	15.6 ± 10.1	<0.001	0.5	20.9 ± 15.3	18.7 ± 12.8	<0.001	0.156

**Table 2:** Hazard ratio of COVID-19 patients with or without DM for pancreas and biliary complications within 90 days.

	Risk (%) DM	Without DM	Hazard Ratio	95% CI	*p*-Value
Pancreatitis					
within 90 days	181/112, 288	139/113, 169	1.307	(1.048, 1.630)	0.017
Cholangitis					
within 90 days	51/115, 543	60/115, 610	0.847	(0.583, 1.230)	0.382
Cholecystitis					
within 90 days	234/113, 029	184/112, 837	1.264	(1.042, 1.533)	0.017
Mortality					
within 90 days	6649/102, 560	5047/102, 549	1.226	(1.183, 1.271)	0.000

## Data Availability

The data that support the findings of this study are derived from the TriNetX research network (https://www.trinetx.com), a global federated health research platform that provides access to de-identified electronic medical records from participating healthcare organizations. Due to data use agreements and ethical restrictions related to patient privacy and confidentiality, the raw data are not publicly available. Researchers interested in accessing similar data may apply directly to TriNetX.

## Abbreviations

The following abbreviations are used in this manuscript:

T2DM	type 2 diabetes mellitus
RR	risk ratio
HR	hazard ratio
OR	odds ratios
CIs	95% confidence intervals

## References

[1] Wiersinga W.J., Rhodes A., Cheng A.C., Peacock S.J., Prescott H.C. (2020). Pathophysiology, Transmission, Diagnosis, and Treatment of Coronavirus Disease 2019 (COVID-19): A Review. JAMA.

[2] Ramos-Casals M., Brito-Zerón P., Mariette X. (2021). Systemic and organ-specific immune-related manifestations of COVID-19. Nat. Rev. Rheumatol..

[3] Umakanthan S., Sahu P., Ranade A.V., Bukelo M.M., Rao J.S., Abrahao-Machado L.F., Dahal S., Kumar H., Kv D. (2020). Origin, transmission, diagnosis and management of coronavirus disease 2019 (COVID-19). Postgrad. Med. J..

[4] Xie N.N., Zhang W.C., Chen J., Tian F.B., Song J.X. (2023). Clinical Characteristics, Diagnosis, and Therapeutics of COVID-19: A Review. Curr. Med. Sci..

[5] Sultan S., Altayar O., Siddique S.M., Davitkov P., Feuerstein J.D., Lim J.K., Falck-Ytter Y., El-Serag H.B. (2020). AGA Institute Rapid Review of the Gastrointestinal and Liver Manifestations of COVID-19, Meta-Analysis of International Data, and Recommendations for the Consultative Management of Patients with COVID-19. Gastroenterology.

[6] Kariyawasam J.C., Jayarajah U., Riza R., Abeysuriya V., Seneviratne S.L. (2021). Gastrointestinal manifestations in COVID-19. Trans. R. Soc. Trop. Med. Hyg..

[7] Han C., Duan C., Zhang S., Spiegel B., Shi H., Wang W., Zhang L., Lin R., Liu J., Ding Z. (2020). Digestive Symptoms in COVID-19 Patients with Mild Disease Severity: Clinical Presentation, Stool Viral RNA Testing, and Outcomes. Am. J. Gastroenterol..

[8] Mutneja H.R., Bhurwal A., Arora S., Goel A., Vohra I., Attar B.M. (2021). Acute pancreatitis in patients with COVID-19 is more severe and lethal: A systematic review and meta-analysis. Scand. J. Gastroenterol..

[9] Onoyama T., Koda H., Hamamoto W., Kawahara S., Sakamoto Y., Yamashita T., Kurumi H., Kawata S., Takeda Y., Matsumoto K. (2022). Review on acute pancreatitis attributed to COVID-19 infection. World J. Gastroenterol..

[10] Bartoli A., Cursaro C., Andreone P. (2022). Severe acute respiratory syndrome coronavirus-2-associated cholangiopathies. Curr. Opin. Gastroenterol..

[11] Faruqui S., Okoli F.C., Olsen S.K., Feldman D.M., Kalia H.S., Park J.S., Stanca C.M., Figueroa Diaz V., Yuan S., Dagher N.N. (2021). Cholangiopathy After Severe COVID-19: Clinical Features and Prognostic Implications. Am. J. Gastroenterol..

[12] Yadlapati S., Jarrett S.A., Baik D., Chaaya A. (2023). COVID-19 related biliary injury: A review of recent literature. World J. Gastroenterol..

[13] Lima-Martínez M.M., Carrera Boada C., Madera-Silva M.D., Marín W., Contreras M. (2021). COVID-19 and diabetes: A bidirectional relationship. Clin. Investig. Arterioscler..

[14] Khunti K., Valabhji J., Misra S. (2023). Diabetes and the COVID-19 pandemic. Diabetologia.

[15] Harding J.L., Oviedo S.A., Ali M.K., Ofotokun I., Gander J.C., Patel S.A., Magliano D.J., Patzer R.E. (2023). The bidirectional association between diabetes and long-COVID-19—A systematic review. Diabetes Res. Clin. Pract..

[16] de-Madaria E., Capurso G. (2021). COVID-19 and acute pancreatitis: Examining the causality. Nat. Rev. Gastroenterol. Hepatol..

[17] Hart P.A., Bradley D., Conwell D.L., Dungan K., Krishna S.G., Wyne K., Bellin M.D., Yadav D., Andersen D.K., Serrano J. (2021). Diabetes following acute pancreatitis. Lancet Gastroenterol. Hepatol..

[18] Urushihara H., Taketsuna M., Liu Y., Oda E., Nakamura M., Nishiuma S., Maeda R. (2012). Increased Risk of Acute Pancreatitis in Patients with Type 2 Diabetes: An Observational Study Using a Japanese Hospital Database. PLoS ONE.

[19] Ludwig R.J., Anson M., Zirpel H., Thaci D., Olbrich H., Bieber K., Kridin K., Dempfle A., Curman P., Zhao S.S. (2025). A comprehensive review of methodologies and application to use the real-world data and analytics platform TriNetX. Front. Pharmacol..

[20] Leis A., Fradera M., Peña-Gómez C., Aguilera P., Hernandez G., Parralejo A., Ramírez-Anguita J.M., Mayer M.A. (2025). Real World Data and Real World Evidence Using TriNetX: The TauliMar Clinical Research Network. Stud. Health Technol. Inform..

[21] Collaborators G.D. (2023). Global, regional, and national burden of diabetes from 1990 to 2021, with projections of prevalence to 2050: A systematic analysis for the Global Burden of Disease Study 2021. Lancet.

[22] Crook H., Raza S., Nowell J., Young M., Edison P. (2021). Long covid-mechanisms, risk factors, and management. BMJ.

[23] Babel R.A., Dandekar M.P. (2021). A Review on Cellular and Molecular Mechanisms Linked to the Development of Diabetes Complications. Curr. Diabetes Rev..

[24] Paul S., Ali A., Katare R. (2020). Molecular complexities underlying the vascular complications of diabetes mellitus—A comprehensive review. J. Diabetes Complicat..

[25] Cheung K.S., Hung I.F.N., Chan P.P.Y., Lung K.C., Tso E., Liu R., Ng Y.Y., Chu M.Y., Chung T.W.H., Tam A.R. (2020). Gastrointestinal Manifestations of SARS-CoV-2 Infection and Virus Load in Fecal Samples From a Hong Kong Cohort: Systematic Review and Meta-analysis. Gastroenterology.

[26] Thomaidou E., Karlafti E., Didagelos M., Megari K., Argiriadou E., Akinosoglou K., Paramythiotis D., Savopoulos C. (2024). Acalculous Cholecystitis in COVID-19 Patients: A Narrative Review. Viruses.

[27] Aziz A.A., Aziz M.A., Omar N., Saleem M., Pahuja K.H., Haseeb Ul Rasool M., Shah R. (2023). A Meta-analysis of the Severity of Acute Pancreatitis (AP) in COVID-19 Infection. Cureus.

[28] Wang F., Wang H., Fan J., Zhang Y., Wang H., Zhao Q. (2020). Pancreatic Injury Patterns in Patients with Coronavirus Disease 19 Pneumonia. Gastroenterology.

[29] Inamdar S., Benias P.C., Liu Y., Sejpal D.V., Satapathy S.K., Trindade A.J. (2020). Prevalence, Risk Factors, and Outcomes of Hospitalized Patients with Coronavirus Disease 2019 Presenting as Acute Pancreatitis. Gastroenterology.

[30] Hartl L., Haslinger K., Angerer M., Semmler G., Schneeweiss-Gleixner M., Jachs M., Simbrunner B., Bauer D.J.M., Eigenbauer E., Strassl R. (2022). Progressive cholestasis and associated sclerosing cholangitis are frequent complications of COVID-19 in patients with chronic liver disease. Hepatology.

[31] Cha M.H., Regueiro M., Sandhu D.S. (2020). Gastrointestinal and hepatic manifestations of COVID-19: A comprehensive review. World J. Gastroenterol..

[32] Cole J.B., Florez J.C. (2020). Genetics of diabetes mellitus and diabetes complications. Nat. Rev. Nephrol..

[33] Tariq Z., Abusnana S., Mussa B.M., Zakaria H. (2024). New insights on genetic background of major diabetic vascular complications. Diabetol. Metab. Syndr..

[34] Pahomeanu M.R., Ojog D., Nițu D.T., Diaconu I.Ș., Nayyerani H., Negreanu L. (2024). Acute Pancreatitis and Type 2 Diabetes Mellitus: The Chicken–Egg Paradox—A Seven-Year Experience of a Large Tertiary Center. J. Clin. Med..

